# 

**Published:** 1871-04

**Authors:** 


### Books and Pamphlets Received.

Surgical Memoirs of the War of the Rebellion, collected and published by the United States Sanitary Commission. Edited by Prof. Frank Hastings Hamilton.

The causation, course and treatment of Reflex Insanity in Women. By Horatio R. Storer, M. D., L. L. B. Boston.

Bound Vols. I. & II. of the Gynæcological Journal. Boston.

Anæsthetics. By Edward R. Squibb, M. D., of Brooklyn, N. Y.

Transactions of the State Medical Society of Michigan.

Woman as a Physician. By J. P. Chesney, M. D.

Rush Medical College. Valedictory Address to the Graduating Class, 1870-1. By Moses Gunn, A. M., M. D., Professor of Surgery.

Report of the Board of Trustees of the Michigan Asylum for the Insane, for the years 1869–1870.

Harvard University Announcement. Medical Department, 1871.

Report of the Resident Physician of Brigham Hall, a Hospital for the Insane, for the year 1870. Canandaigua, N. Y.

Eleventh Annual Report of the Superintendent of the State Lunatic Asylum for Insane Criminals at Auburn, N. Y.

The Freeman Trial. By David Dimond, M. D. Auburn, N. Y.

Management of the Obstetrical Forceps. By C. C. P. Clark, M. D. Oswego, N. Y.

